# Supplementation with a Cocoa–Carob Blend, Alone or in Combination with Metformin, Attenuates Diabetic Cardiomyopathy, Cardiac Oxidative Stress and Inflammation in Zucker Diabetic Rats

**DOI:** 10.3390/antiox11020432

**Published:** 2022-02-21

**Authors:** Esther García-Díez, María Elvira López-Oliva, Alicia Caro-Vadillo, Francisco Pérez-Vizcaíno, Jara Pérez-Jiménez, Sonia Ramos, María Ángeles Martín

**Affiliations:** 1 Instituto de Ciencia y Tecnología de Alimentos y Nutrición (ICTAN-CSIC), 28040 Madrid, Spain; esther.garciad@ictan.csic.es (E.G.-D.); jara.perez@ictan.csic.es (J.P.-J.); s.ramos@ictan.csic.es (S.R.); 2 Departamento de Fisiología, Facultad de Farmacia, Universidad Complutense de Madrid, 28040 Madrid, Spain; elopez@farm.ucm.es; 3 Departamento de Medicina y Cirugía Animal, Facultad de Veterinaria, Universidad Complutense de Madrid, 28040 Madrid, Spain; aliciac@vet.ucm.es; 4 Departamento de Farmacología y Toxicología, Facultad de Medicina, Universidad Complutense de Madrid, 28040 Madrid, Spain; fperez@med.ucm.es; 5 Instituto de Investigación Sanitaria Gregorio Marañón (IISGM), 28007 Madrid, Spain; 6 CIBER de Enfermedades Respiratorias (CIBERES), Instituto de Salud Carlos III, 28029 Madrid, Spain; 7 CIBER de Diabetes y Enfermedades Metabólicas Asociadas (CIBERDEM), Instituto de Salud Carlos III, 28029 Madrid, Spain

**Keywords:** heart, flavonoids, diabetes, antioxidants, metformin, SIRT1, Nrf2

## Abstract

Diabetic cardiomyopathy (DCM) is one of the main causes of mortality among diabetic patients, with oxidative stress and inflammation major contributors to its development. Dietary flavonoids show strong antioxidant and anti-inflammatory activities, although their potential additive outcomes in combination with antidiabetic drugs have been scarcely explored. The present study investigates the cardioprotective effects of a cocoa–carob blend (CCB) diet, rich in flavonoids, alone or in combination with metformin, in the development of DCM. Zucker diabetic fatty rats (ZDF) were fed with a CCB rich-diet or a control diet, with or without metformin for 12 weeks. Glucose homeostasis, cardiac structure and function, and oxidative and inflammatory biomarkers were analysed. CCB improved glucose homeostasis, and mitigated cardiac dysfunction, hypertrophy, and fibrosis in ZDF rats. Mechanistically, CCB counteracted oxidative stress in diabetic hearts by down-regulating NADPH oxidases, reducing reactive oxygen species (ROS) generation and modulating the sirtuin-1 (SIRT1)/ nuclear factor E2-related factor 2 (Nrf2) signalling pathway, overall improving antioxidant defence. Moreover, CCB suppressed inflammatory and fibrotic reactions by inhibiting nuclear factor kappa B (NFκB) and pro-inflammatory and pro-fibrotic cytokines. Noteworthy, several of these effects were further improved in combination with metformin. Our results demonstrate that CCB strongly prevents the cardiac remodelling and dysfunction observed in diabetic animals, highlighting its potential, alone or in adjuvant therapy, for treating DCM.

## 1. Introduction

Type 2 diabetes (T2D) represents a major worldwide health problem because of its high prevalence and associated cardiovascular complications [1]. Among them, diabetic cardiomyopathy (DCM) is one of the main causes of diabetes-associated morbidity and mortality [2]. There are many glucose-lowering drugs available for the treatment of T2D. Some of them may reduce the cardiovascular mortality but the risk of cardiovascular death is still very high in this population. Therefore, development of novel preventive or therapeutic strategies is needed to treat diabetic cardiomyopathy.

DCM is defined as the existence of abnormal myocardial structure and function in the heart of diabetic patients in the absence of other cardiac risk factors [3]. Oxidative stress is considered one of the most important contributors to the pathogenesis of DCM [4]. Thus, the overproduction of reactive oxygen species (ROS) in diabetic hearts activates several detrimental pathways, including inflammation, hypertrophy, fibrosis, and cell death that eventually induce irreversible structural remodelling and dysfunction of this organ [5]. At the molecular level, hyperglycaemia directly induces the activity of NADPH oxidases (NOX) in cardiomyocytes, resulting in an excessive generation of ROS [6]. At the same time, the expression levels of the nuclear factor-erythroid 2-related factor 2 (Nrf2), involved in the cellular antioxidant defence system, and the levels of sirtuin1 (SIRT-1), a NAD-dependent histone deacetylase that protect against ROS-mediated oxidative damage, are down-regulated in diabetes, worsening the oxidative stress situation in the heart [7,8]. In addition, this pro-oxidant environment in the cardiomyocytes may initiate inflammatory responses via activation of nuclear factor kappa B (NF-κB) signalling, resulting in the up-regulation of several inflammatory cytokines and the induction of profibrotic factors, which contribute to myocardial fibrosis and collagen deposition [9]. Considering this, agents that target both oxidative stress and inflammation are considered very promising candidates to slow down the occurrence of DCM.

In the last years, natural products such as polyphenols have received widespread attention as a preventive approach to alleviate several cardiovascular diseases without adverse side effects [10,11,12]. In addition, the combination of pharmaceutical drugs and natural health substances with synergistic interactions could provide a way to make a more effective treatment [13]. Flavonoids are one of the most abundant group of polyphenols in the human diet and they have been widely investigated for their beneficial effects in several cardiovascular pathologies [14]. More recently, the study of the potential of dietary flavonoids or flavonoid rich diets during DCM is emerging. It has been indicated that flavonoids have the potential to alleviate DCM by their anti-hyperglycaemic, antioxidant and anti-inflammatory properties [15]. In this regard, cocoa flavonoids could be considered very good candidates for DCM treatment since they possess antioxidants and antidiabetic properties [16,17], as well as favourable cardiovascular effects [18,19,20]. However, cocoa is characterised by a bitter taste, rejected by many people. This taste is often modified by adding sugars, thus generating products with a worse nutritional profile. In contrast, carob (*Ceratonia siliqua* L.) bean, a Mediterranean legume rich in polymeric flavanols and other polyphenol classes, has proven to be a good option to be combined with cocoa [21], generating mixtures with a high phytochemical content. Indeed, carob is being increasingly studied for its potential beneficial effects, particularly regarding glucose homeostasis [22,23,24].

In view of this, we have developed a potential functional food combining cocoa powder with carob flour (in a proportion 60:40) for obtaining a cocoa–carob blend (CCB) rich in polyphenols (particularly non-extractable proanthocyanidins) with decreased bitterness (Garcia-Diez et al., submitted). The aim of this study was to investigate whether this functional food, rich in flavonoids, may prevent the development of DCM in an animal model of T2D. The potential additive effect of the CCB supplementation in combination with metformin, the first line anti-hyperglycaemic agent for the treatment of T2D, was evaluated as well. Our results provide strong evidence for the beneficial impact of the functional CCB rich-diet, alone or in combination with metformin, against the development of cardiac remodelling and dysfunction in diabetic animals and suggest the possible mechanisms involved in this protection.

## 2. Materials and Methods

### 2.1. Materials and Chemicals

Nicotine adenine dinucleotide reduced salt (NADPH), 2′,7′-dichlorofluorescin diacetate (DCFH), reduced glutathione (GSH), glutathione reductase (GR), *tert*-butylhydroperoxide, o-phthaldehyde, streptavidin-biotin conjugated horseradish peroxidase (HRP), 3, 3′-diaminobenzidine (DAB) and glucose were purchased from Sigma Chemical (Madrid, Spain). Anti-TFGβ1 (sc-52893), anti-NADPH oxidase (NOX)-4 (sc-30141), anti-SIRT1 (sc-74465), anti-phospho-Ser536-p65-NFκB (sc-135769) anti-TNFα (sc-52746), anti-interleukin (IL)-6 (sc-57315) and anti-monocyte chemoattractant protein-1 (MCP-1) (sc-52701) were purchased from Santa Cruz Biotechnology (Quimigen, Madrid, Spain). Antiphospho-Nrf2 (#12811) was purchased from Signalway antibody (Quimigen, Madrid, Spain). Anti-NOX2 (ab-80897) and anti-CD45 (ab-10558), were purchased from Abcam (Cambrigde, UK). Terminal Transferase recombinant, biotin-16-dUTP and proteinase K were from Roche Applied Science (Roche Diagnostic, Barcelona, Spain). Bradford reagent was from BioRad (BioRad Laboratories S.A., Madrid, Spain).

### 2.2. Cocoa–Carob Blend Diet

Cocoa–carob blend (CCB) was prepared by mixing pure cocoa powder (a kind gift from Idilia S.L., Barcelona, Spain) with carob flour (Casa Ruiz Granel Selecto S.L., Madrid, Spain) in a proportion of 60:40. The product was characterized by a high polyphenol and dietary fibre content (16.7 and 55.7 g/100 g, respectively). Among polyphenols, most of them (12.1% in CCB) were non-extractable proanthocyanidins, i.e., high molecular weight flavanols mostly associated with dietary fibre. Regarding dietary fibre, it was mostly insoluble dietary fibre (51% in CCB). Both cocoa and carob contributed with characteristic substances, as shown by chromatography analysis: thus, CCB contained theobromine (23 mg/100 g) from cocoa and D-pinitol (1.22%) derived from carob. A detailed characterization of CCB has been provided elsewhere (García-Díez et al., submitted). CCB rich-diet (10%) was produced by adding 100 g/kg of the CCB to the AIN-93G diet. The resulting CCB diet was isoenergetic and its composition is given in Table 1.

### 2.3. Animals and Experimental Design

Male Zucker diabetic fatty (ZDF, *n* = 32) rats and their Zucker lean controls (ZL, *n* = 6) were purchased from Charles River Laboratories (L’arbresle, France) at 11 weeks of age. Animals were acclimated for one week under standard controlled conditions (21 ± 1 °C; 50–60% humidity 12 h day/night cycle). Afterwards, ZDF diabetic rats (12 weeks of age) were randomly sorted into four different experimental groups of eight animals: (1) ZDF diabetic rats received a standard AIN-93G diet (ZDF); (2) ZDF rats received a standard AIN-93G diet and oral metformin (300 mg/kg/day) (ZDF (M)); (3) ZDF rats received the CCB diet (ZDF (CCB)) and 4) ZDF rats received the CCB diet and metformin (300 mg/kg/day) (ZDF (CCB + M)). The lean Zucker rats (ZL) received the standard AIN-93G diet. Drinking water served as the vehicle for metformin treatment of ZDF rats. During the study period (12 weeks), all experimental groups were provided with food and water *ad libitum*. Food and water intakes were monitored daily, and body weight and postprandial blood glucose were followed weekly during the entire study. At 24 weeks of age, animals were fasted overnight and sacrificed. Blood samples were collected for biochemical analysis, hearts were rapidly resected, and the left ventricles (LV) excised for the different analyses.

Animals were treated according to the European (2010/63/EU) and Spanish (RD 53/2013) legislation on Care and Use of Experimental Animals and the experiments were approved by the Ethics Committee from Comunidad de Madrid (PROEX 079/19).

### 2.4. Biochemical Determination

Fasting blood samples were collected for glucose, insulin, glycosylated haemoglobin (Hb1Ac), triacylglycerols (TG), HDL, and LDL analysis. Blood glucose was determined using an Accounted Glucose Analyser (LifeScan España, Madrid, Spain). Serum insulin and Hb1Ac were analysed with an ELISA kit (Rat Insulin, Mercodia, Uppsala, Sweden; HbA1c Kit Spinreact, BioAnalitica, Madrid, Spain). Fasting glycaemia and insulinaemia were used to calculate indices of homeostatic model assessment of insulin resistance (HOMA-IR) and secretion (HOMA-B) according to the following formulas: HOMA-IR = fasting insulin (mU/mL) × fasting glucose (mM)/22.5 and HOMA-B = 20 × fasting insulin (mU/mL)/[fasting glucose (mM)-3.5], respectively. TG, T-Cho, HDL-Cho, and LDL-Cho were determined in serum by kits (BioSystems, Madrid, Spain).

### 2.5. Glucose Tolerance Test

One week before the sacrifice, a glucose tolerance test (GTT) was performed. After overnight fasting, 35% glucose solution (2 g/kg of body weight) was administrated to rats by intraperitoneal injection and blood samples were obtained from the tail vein before the glucose load (t = 0) and at, 30, 60, 90 and 120 min after glucose administration. The measures were determined with a glucometer. Changes in glucose were calculated as the area under the curve (AUC) above the basal levels.

### 2.6. Echocardiographic Measurements

Cardiac function was evaluated by echocardiography. Echocardiographic studies were performed in the last week of the experiments using the General Electric Vivid iq (GE Medical Systems, Changjiang, China) echocardiographic system, equipped with a 8 to 18 MHz linear probe. Images were obtained from animals lightly anesthetized with 1–2% isoflurane (IsoFlo, Abbot Laboratories, Madrid, Spain). Left ventricular (LV) end-diastolic dimensions (LVDD), LV end-systolic dimensions (LVSD), intraventricular septum diastolic diameter (IVSD), LV posterior wall diastolic diameter (LVPWD), left atrial diameters (LA), aortic dimensions (Ao), left ventricular ejection fraction (EF) and LV fractional shortening (FS) were measured following the American Society for Echocardiography’s leading-edge method [25]. All images were digitally acquired and analysed by an experienced sonographer unaware of the treatment group allocation.

### 2.7. Histological and Immunohistochemical Analyses

Left ventricles were rinsed with phosphate-buffered saline (PBS), fixed in 4% paraformaldehyde at 4 °C overnight, embedded in paraffin and cut into 5 μm thick sections. For the morphometric analysis, LV sections were stained with haematoxylin and eosin (H and E) and the extent of cardiac myocyte hypertrophy was measured by cross sectional area (CSA) determination. Cardiomyocyte CSA was measured by manually outlining 120 cells per heart in HE-stained sections using ImageJ. At 400-fold magnification, seven sequential, nonoverlapping fields of view were scanned to represent the whole cross section. Ten cardiomyocytes per field, transversely sectioned, with a round shape and visible central nucleus, and located in the subendocardial layer of the LV muscle wall were selected.

For the quantification of fibrosis, serial sections of LV samples were stained with Masson’s trichrome, which stains the cardiomyocytes red and the fibrosis blue. Each section was examined in 5 randomly selected high-power fields (×400, ×630) and middle-power fields (×200) under a light microscope. Percent fibrosis area was analysed using ImageJ analysis software (Version 1.8; National Institutes of Health) and expressed as percentage of collagen area (blue) to the total area of each microscopic field.

For the immunohistochemical staining, antibodies against TFGβ1, CD45, NOX2, NOX4, SIRT1, p-Nrf2, p-p65-NFκB, TNFα, IL6 and MCP1 were used. Serial left ventricular sections were incubated with the primary antibodies overnight at 4 °C, then incubated with HRP-conjugated secondary antibody and revealed with DAB substrate as chromogen and haematoxylin. TFGβ1, NOX2, NOX4, SIRT1, p-Nrf2, TNFα, IL6 and MCP1 expression was evaluated by their staining pattern: weak (1), moderate (2), diffuse (3) and intense (4). The percentages of p-p65-NFκB positive nuclei were calculated as the number of positive nuclei × 100/total number of nuclei. The percentages of CD45 positive cells were calculated as the number of positive cell × 100/total number of cells. Six sections per group were analysed.

### 2.8. Determination of ROS

ROS were quantified by the dichlorofluorescein (DCFH) assay based on the oxidation of dichlorofluorescein (DCF) that emits fluorescence [19]. LV homogenates were diluted with ice-cold Locke’s buffer (154 mM NaCl, 5.6 mM, KCl, 3.6 mM NaHCO_3_, 2 mM CaCl_2_, 10 mM D-glucose and 5 mM 4-(2-hydroxyethyl)-1-piperazineethanesulfocnic acid, pH 7.4) and incubated with 5 μM DCFH for 30 min at 37 °C in darkness. Fluorescence was measured in a microplate reader (Bio-Tek, Winooski, VT, USA) at an excitation wavelength of 485 nm and an emission wavelength of 530 nm. Proteins in LV homogenates were measured by using the Bradford reagent.

### 2.9. Determination of Protein Carbonyl Content

Protein oxidation of LV homogenates was measured as carbonyl levels [26]. Samples were homogenized in 0.25 M Tris (pH 7.4), 0.2 M sucrose and 5 mM 1,4-dithiothreitol (DTT) buffer and derivatized with 2,4 dinitrophenylhydrazine (DNPH). Absorbance was measured at 360 nm and carbonyl content was expressed as nmol/mg protein using an extinction coefficient of 22,000 nmol L^−1^ cm^−1^. Protein in LV homogenates was determined by the Bradford reagent.

### 2.10. GSH Levels and GPx and GR Activities

The concentration of GSH was evaluated by a fluorometric assay already described in [26]. The method considers the reaction of GSH with o-phthalaldehyde (OPT) at pH 8.0. The LV samples were homogenized in 50 mM phosphate buffer pH 7.0, and proteins were precipitated with 5% trichloroacetic acid and then centrifuged for 30 min at 10,000× *g*. The fluorescence was measure at 460 nm—emission wavelength—and at 340 nm—excitation wavelength. For determining the activity of antioxidant enzymes (GPx and GR), LV samples were homogenized in 0.25 M Tris, 0.2 M sucrose and 5 mM DTT buffer pH 7.4 and centrifuged at 3000× *g* for 15 min. GPx activity was assayed by the oxidation of GSH by GPx, where *tert*-butylhydroperoxide (*t*-BOOH) is the substrate, coupled to the disappearance of NADPH by GR [26]. GR activity was determined by following the decrease in absorbance due to the oxidation of NADPH utilized in the reduction of oxidized glutathione [26].

### 2.11. Statistical Analysis

Statistical analysis was performed using the GraphPad Prism 6 software package. Gaussian distribution was assessed with the Shapiro–Wilk normality test. Parametric data were tested with one-way analyses of variance (ANOVA), while the Kruskal–Wallis test was performed for non parametric data (echocardiographic parameters). Statistically significant differences between means were determined using the Tukey and the Mann–Whitney post-hoc tests. A two-tailed *p* < 0.05 was considered significant. All data are expressed as mean ± standard deviation.

## 3. Results

### 3.1. Effect of CCB Alone or in Combination with Metformin on Glucose Homeostasis and Lipid Profile in Zucker Diabetic Rats

As observed in Table 2, initially Zucker diabetic rats exhibited a higher body weight compared to non-diabetic ZL animals, confirming their obesity. At the end of the study, the final body weight was similar between ZL and ZDF animals, except for (ZDF (M)) group that showed a slightly but significantly higher body weight; this was indicative of the ZDF rat diabetic status. The total food intake during the 12 weeks of the study was higher in all ZDF groups as compared to ZL animals; therefore, the food efficiency was significantly decreased in ZDF animals.

**Figure 1:**
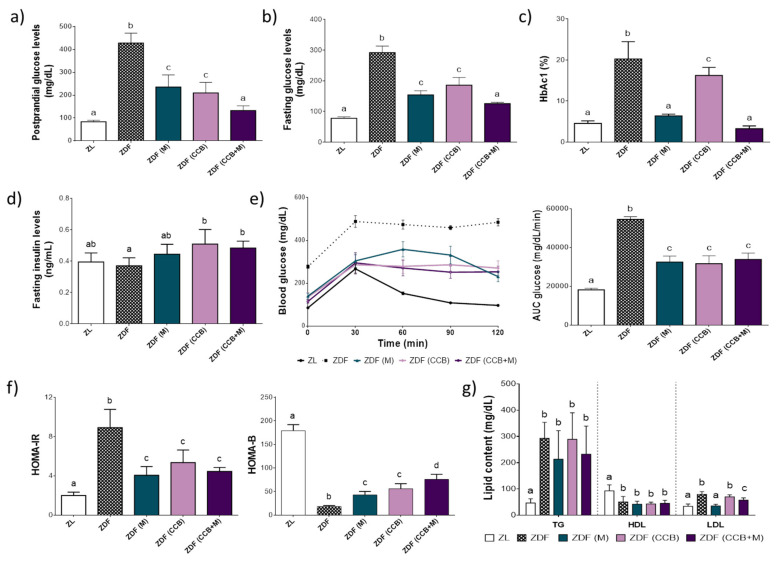
Effect of CCB, metformin and their combination on glucose and lipid homeostasis. (**a**) Postprandial glucose levels. (**b**) Fasting glucose levels. (**c**) Glycosylated haemoglobin (HbA1c). (**d**) Serum insulin levels. (**e**) Blood glucose levels during GTT and area under the curve (AUC) calculated from the GTT data. (**f**) Homeostasis model assessment (HOMA)-IR and HOMA-B. (**g**) Levels of TG, HDL, and LDL in serum. Data represent the means ± SD of 6–8 animals per condition. Means without a common letter differ, *p* < 0.05. Zucker lean (ZL), Zucker Diabetic rats (ZDF), Zucker Diabetic rats treated with metformin (ZDF (M)), Zucker Diabetic rats fed with a CCB rich-diet (ZDF (CCB)), and Zucker Diabetic rats treated with metformin and fed with a CCB rich-diet (ZDF (CCB + M)).

Regarding glucose homeostasis, at the end of the study, ZDF control rats evidenced a significant increase in fasting and postprandial glucose levels, as well as in the percentage of glycosylated haemoglobin (Hb1Ac) as compared to non-diabetic ZL rats, while insulinaemia was similar in both populations (Figure 1a–d). Furthermore, ZDF rats showed glucose intolerance (measured by AUC), increased insulin resistance (HOMA-IR) and decreased pancreatic function (HOMA-B) (Figure 1e,f), which further confirmed their diabetic state. Treatment with metformin (ZDF (M)) or the CCB diet (ZDF (CCB)) for 12 weeks significantly improved all these parameters. Besides, animals fed with the CCB diet and treated with metformin (ZDF (CCB + M)) achieved values of both postprandial and fasting glucose and HbAc1 similar to those found in the non-diabetic rats (ZL). On the contrary, none of the treatments could improve the increase in TG and HDL levels in diabetics, and only the metformin treatment prevented the increase in LDL levels in ZDF animals (Figure 1g).

These results indicate that a CCB rich diet significantly improved glucose homeostasis in ZDF rats. Notably, combining treatment with metformin had a superior effect on glucose control in comparison to the agents alone.

### 3.2. Effect of CCB, Metformin and Their Combination on Cardiac Function in Zucker Diabetic Rats

**Figure 2:**
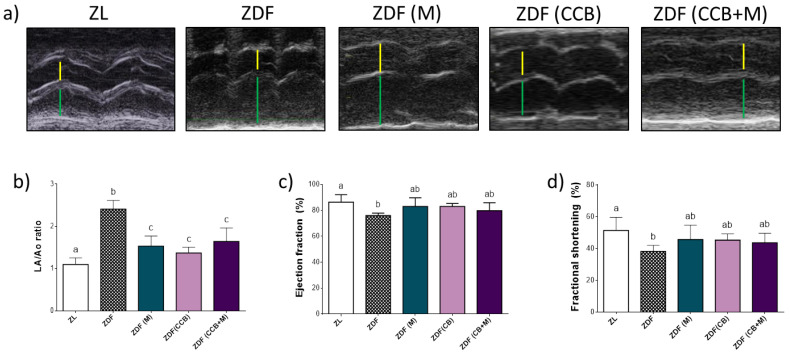
Effect of CCB, metformin and their combination on cardiac function. (**a**) Representative images of M-mode echocardiogram for each group with the measurement points of aorta diameters (yellow lines) and left atrium diameter (green lines). (**b**) LA/Ao ratio. (**c**) LV ejection fraction (EF). (**d**) LV fractional shortening (FS). Means ± SD of 6–8 samples per condition. Means without a common letter differ significantly. Zucker lean (ZL), Zucker Diabetic rats (ZDF), Zucker Diabetic rats treated with metformin (ZDF (M)), Zucker Diabetic rats fed with a CCB rich-diet (ZDF (CCB)), and Zucker Diabetic rats treated with metformin and fed with a CCB rich-diet (ZDF (CCB + M)).

At 24 weeks old, the heart weight-to-tibia length ratio (HW/TL) was similar between groups being significantly decreased only in ZDF animals fed with the CCB rich-diet (ZDF (CCB)) (Table 3). To evaluate the effect of metformin and the CCB diet on cardiac function we performed echocardiography. Data indicated that left ventricular end-diastolic dimensions (LVDD), left ventricular end-systolic dimensions (LVSD), septum diastolic diameter (IVSD) and LV posterior wall diastolic diameter (LVPWD) did not differ among groups. However, a significantly increase in the left atrium diameter (LA) and a decrease in the aorta diameter (Ao) were observed in control diabetic rats (ZDF) compared to non-diabetic animals (ZL) (Table 3 and Figure 2a). Consequently, the LA/Ao ratio, which is an indicator of LA enlargement and remodelling, was significantly increased in ZDF control rats (Figure 2b). Importantly, treatment of diabetic rats with metformin and the CCB rich-diet significantly reduced this ratio with no additional effect of the combination. Likewise, LV ejection fraction (EF) and LV fractional shortening (FS) were slightly but significantly decreased in the ZDF control group, suggesting initial systolic dysfunction in these diabetic animals (Figure 2c,d). This detrimental effect was prevented in all other animal groups receiving metformin and CCB alone or in combination. Taken together, these data suggested that both metformin and the CCB diet could alleviate cardiac dysfunction in the left ventricle of diabetic rats.

### 3.3. Effect of CCB, Metformin and Their Combination on LV Remodelling in Zucker Diabetic Rats

**Figure 3:**
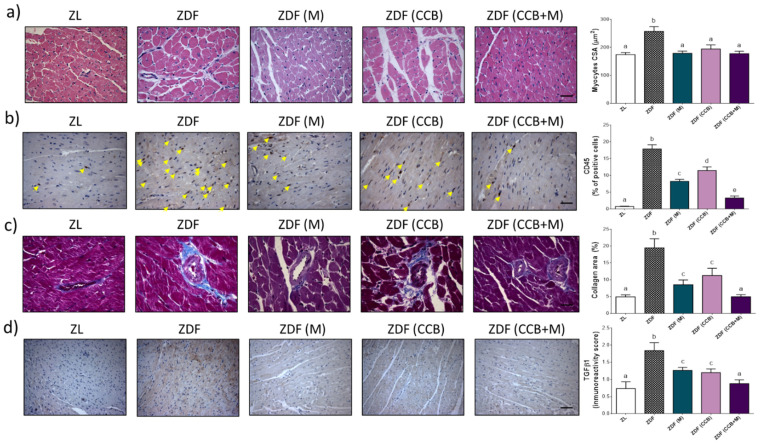
Effect of CCB, metformin and their combination on cardiac remodelling. (**a**) Representative sections of left ventricles stained with H and E (scale bars: 10μm) and quantitative data for myocyte cross sectional area. (**b**) Representative photographs of immunohistochemical staining of CD45 (brown-stained) (scale bar 10 μm). CD45 is expressed as a percentage of positive cells relative to total cells. (**c**) Representative images of collagen fibres in interstitial and perivascular areas shown by Masson’s trichrome staining (blue-stained) (scale bars: 10μm) and quantification of collagen areas (%). (**d**) Representative photographs of immunohistochemical staining of TFG-β1 (brown-stained) (scale bar 20 μm) and immunoreactive score. Values are expressed as means ± SD of 6–8 animals per condition. Means without a common letter differ, *p* < 0.05. Zucker lean (ZL), Zucker Diabetic rats (ZDF), Zucker Diabetic rats treated with metformin (ZDF (M)), Zucker Diabetic rats fed with a CCB rich-diet (ZDF (CCB)), and Zucker Diabetic rats treated with metformin and fed with a CCB rich-diet (ZDF (CCB + M)).

Since cardiac dysfunction results from alterations in the composition and structure of the heart, we then investigated structural changes and remodelling in the LV of diabetic animals. Results from H and E-staining of the LV tissues (Figure 3a) revealed that cardiomyocytes were arranged in neat rows with uniform size nuclei in the ZL non-diabetic group. However, in the ZDF control animals, the myocardial fibres were disordered, and the cardiomyocytes were swollen and enlarged significantly, displaying an uneven cytoplasm and eccentric nucleus. In addition, the myocardial gap was widened, demonstrating marked perivascular and interstitial oedema. Likewise, the levels of CD45 (marker of immune cell infiltration) were significantly increased in the myocytes of ZDF diabetic control rats (Figure 3b). Administration of metformin or CCB and their combination, strongly prevented these degenerative changes with appearance of the classical healthy cardiomyocytes. Cardiomyocyte hypertrophy in diabetic rats (ZDF) was evidenced by increased cardiomyocyte cross-sectional area (CSA), while diabetic animals treated with metformin (ZDF (M)), fed with the CCB-diet (ZDF (CCB)) or both (ZDF (CCB + M)) presented a normal cell size in comparison to the diabetic group. Next, to detect fibrosis and collagen content, Masson’s trichrome staining was performed in LV tissues. Likewise, the expression of a fibrotic marker, the transforming growth factor β1 (TGFβ1), was determined. As observed in Figure 3c,d, interstitial and perivascular collagen fractional area and the expression levels of TGFβ1 were significantly increased in the left ventricles from ZDF diabetic rats as compared to ZL animals. All these adverse structural modifications induced by diabetes were partially prevented with metformin (ZDF (M)) or the CCB diet (ZDF (CCB)), with a further significant reduction by the combination (ZDF (CCB + M)). Indeed, the CCB rich-diet combined with metformin totally prevented myocardial cellular damage (hypertrophy and fibrosis) in the LV of diabetic animals.

### 3.4. Effect of CCB, Alone or in Combination with Metformin on Cardiac Oxidative Stress in Zucker Diabetic Rats

**Figure 4:**
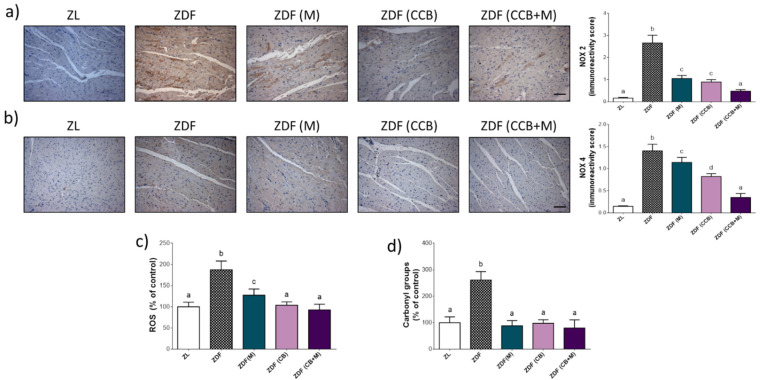
Effect of CCB, metformin and their combination on redox status. (**a**) Representative photographs of immunohistochemical staining of NOX2 (brown-stained) (scale bar 20 μm) and immunoreactivity score. (**b**) Representative photographs of immunohistochemical staining of NOX4 (brown-stained) (scale bar 20 μm) and immunoreactivity score. (**c**) Percentage levels of ROS relative to the control condition. (**d**) Percentage levels of carbonyl groups relative to the control condition (ZL). Means ± SD of 6–8 samples per condition. Means without a common letter differ significantly. Zucker lean (ZL), Zucker Diabetic rats (ZDF), Zucker Diabetic rats treated with metformin (ZDF (M)), Zucker Diabetic rats fed with a CCB rich-diet (ZDF (CCB)), and Zucker Diabetic rats treated with metformin and fed with a CCB rich-diet (ZDF (CCB + M)).

Oxidative stress is a principal mediator of DCM; we next investigated the oxidative status of the LV of the diabetic animals. To this end, we evaluated the levels of NOX2 and NOX4, the main enzymes involved in the production of ROS induced by hyperglycaemia. As showed in Figure 4a–c, the levels of NOX2 and NOX4 and ROS generation were significantly increased in the left ventricle of diabetic control rats (ZDF). Metformin (ZDF (M)) and the CCB rich-diet (ZDF (CCB)) significantly reduced NOX2 and NOX4 protein expressions and decreased cardiac ROS levels, whilst the combination of the CCB rich-diet with metformin (ZDF (CCB + M)) totally avoided the oxidative stress in diabetic animals. As a result, oxidative injury, indicated by protein carbonyl levels (Figure 4d), was noted in diabetic control rats (ZDF) but not in those treated with metformin (ZDF (M)), fed with the CCB diet (ZDF (CCB)) or both (ZDF (CCB + M)).

### 3.5. Effect of CCB and Their Combination with Metformin on SIRT/Nrf2 Pathway and Antioxidant Response in Zucker Diabetic Rats

**Figure 5:**
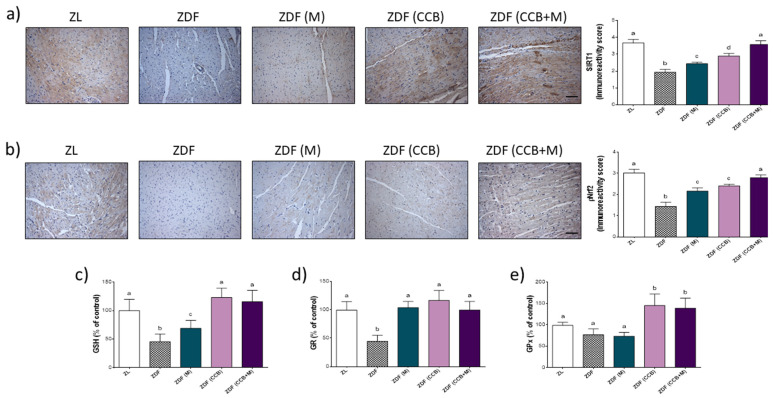
Effect of CCB, metformin and their combination on SIRT1, Nrf2 and main antioxidant defences. (**a**) Representative photographs of immunohistochemical staining of SIRT1 (brown-stained) (scale bar 20 μm) and immunoreactivity score. (**b**) Representative photographs of immunohistochemical staining of p-Nrf2 (brown-stained) (scale bar 20 μm) and immunoreactive score. (**c**) Percentage levels of GSH relative to the control condition. (**d**) Percentage levels of GR activity relative to the control condition. (**e**) Percentage levels of GPx activity relative to the control condition. Data represent means ± SD of 6–8 samples per condition. Means without a common letter differ significantly, *p* < 0.05. Zucker lean (ZL), Zucker Diabetic rats (ZDF), Zucker Diabetic rats treated with metformin (ZDF (M)), Zucker Diabetic rats fed with a CCB rich-diet (ZDF (CCB)), and Zucker Diabetic rats treated with metformin and fed with a CCB rich-diet (ZDF (CCB + M)).

Since SIRT1 and Nrf2 could reduce myocardial cell oxidative stress and modulate the antioxidant response system in DCM, the levels of both proteins and antioxidant defences were also evaluated in LV tissues. As depicted in Figure 5a,b, the expression of SIRT1 and Nrf2 were prominently decreased in the myocytes of diabetic control rats (ZDF) compared with the ZL non-diabetic group. The treatment with metformin (ZDF (M)) or the CCB diet (ZDF (CCB)) partially prevented these effects, whereas they were totally reversed with the combination (ZDF (CCB + M)). Likewise, the levels of GSH and the activity of the antioxidant enzyme GR were significantly reduced in ZDF control rats but not in those treated with metformin, fed with the CCB diet, or both (Figure 5c,d). Noteworthy, the CCB diet alone (ZDF (CCB)) or in combination with metformin (ZDF (CCB + M)) induced the activity of GPx above the control levels (Figure 5e). Taken together, these outcomes strongly indicate that both metformin and the CCB diet partly prevented diabetes-induced depletion of SIRT1 and Nrf2 and their down-stream antioxidant defences, whilst all these effects were prevented with the combination of CCB with metformin.

### 3.6. Effect of CCB, Metformin and Their Combination on Myocardial Inflammation in Zucker Diabetic Rats

**Figure 6:**
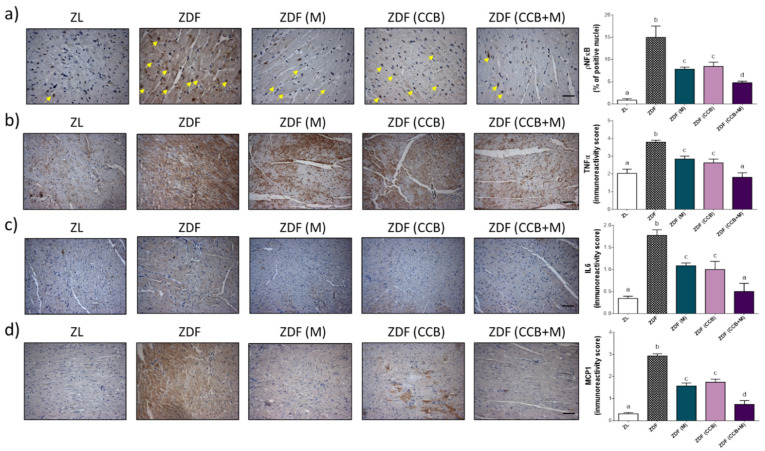
Effect of CCB, metformin and their combination on cardiomyocyte inflammation. (**a**) Representative photographs of immunohistochemical staining of p-NFĸB (brown-stained) (scale bar 10 μm). P-NFĸB is expressed as percentage of positive nucleus relative to total nuclei. (**b**) Representative photographs for immunohistochemical staining of TNFα (brown-stained) (scale bar 20 μm) and immunoreactive score. (**c**) Representative photographs of immunohistochemical staining of IL-6 (brown-stained) (scale bar 20 μm) and immunoreactive score. (**d**) Representative photographs of immunohistochemical staining of MCP1 (brown-stained) (scale bar 20 μm) and immunoreactive score. Values are expressed as mean ± SD (*n* = 6–8). Means without a common letter differ significantly, *p* < 0.05. Zucker lean (ZL), Zucker Diabetic rats (ZDF), Zucker Diabetic rats treated with metformin (ZDF (M)), Zucker Diabetic rats fed with a CCB rich-diet (ZDF (CCB)), and Zucker Diabetic rats treated with metformin and fed with a CCB rich-diet (ZDF (CCB + M)).

Oxidative stress can also induce the release of pro-inflammatory cytokines through the activation of NFκB in cardiac cells. Therefore, we later examined the effect of metformin and CCB in myocardial inflammation. Figure 6a, shows a marked increase in the nuclear levels of p-NFκB in ZDF diabetic control rats. Furthermore, the level of pro-inflammatory cytokines including TNFα, IL6, and MCP1 were significantly increased in the myocytes of ZDF diabetic control rats (Figure 6b–d). Both metformin (ZDF (M)) and the CCB rich-diet (ZDF (CCB)) were able to significantly decrease the NF-κB activation and the expression levels of pro-inflammatory cytokines in the diabetic myocardium. Once again, the combined administration of the CCB rich-diet with metformin (ZDF (CCB + M)) achieved superior protective effects.

## 4. Discussion

Many flavonoids have demonstrated protective roles against several cardiovascular diseases due to their antioxidant and anti-inflammatory activities, and other beneficial functions [14,15]. Moreover, increasing literature suggests synergistic effects when flavonoids are used in combination with each other [27,28]. However, most of the studies used purified flavonoids; research focus on their combination in the DCM model is lacking. Moreover, the effect of their combination with antidiabetic drugs has scarcely been explored. In the present study, we showed, for the first time, the cardioprotective effect of a mix of cocoa and carob flavonoids (CCB), against diabetic cardiomyopathy and its more favourable effect in combination with metformin, the first-line drug used in diabetes.

To investigate the protective action of the CCB product, the Zucker diabetic fatty rats, a well-established animal model of T2D, were used. During the development of the illness, ZDF rats present hyperglycaemia, oxidative stress, and cardiovascular damage, making this model highly appropriate to evaluate the consequence of the CCB rich-diet in the initial stage of DCM. Chronic supplementation with CCB significantly ameliorated fasting and postprandial hyperglycaemia, glucose intolerance and insulin resistance in diabetic animals and these effects were similar to those obtained just with metformin, used as a reference. These results agree with previous reports showing the potential antidiabetic effects of cocoa [16,17] and carob [22,23,24] and support the relevance of flavonoid rich-diets for controlling metabolic disorders such as diabetes [29]. Indeed, foods rich in flavonoids contain many bioactive compounds with multiple targets, which can exert synergistic effects, and, therefore, have the potential to be effective in restoring the multiple molecular pathways altered in T2D [13]. Accordingly, carob composition is complementary to that of cocoa, in terms of phytochemical constituents. Thus, both are rich in polymeric flavanols, mostly associated with dietary fibre, but cocoa also contains methylxanthines (theobromine and caffeine) [30], and carob is rich in other polymeric phenolic structures (gallotannins and ellagitannins) [31]. Besides, carob contains D-pinitol, a polyalcohol for which several biological activities regarding cardiometabolic alterations have been suggested [32]. Therefore, this pool of bioactive substances may eventually exert a beneficial effect on the T2D situation due to a combination of diverse mechanisms of action. Moreover, it should be highlighted that the combination of the CCB rich-diet with metformin was more effective than metformin alone in improving glycaemic control, further demonstrating the effectiveness of combining natural bioactive compounds with antidiabetic drugs in the treatment of diabetes [13]. Especially, improvement of glucose homeostasis by the CCB diet may contribute, at least in part, to attenuate the development of DCM in ZDF rats.

Cardiac dysfunction associated with myocardial fibrosis and hypertrophy is one of the most important characteristics in the progression of DCM [33,34]. In the present study, ZDF animals showed cardiac dysfunction at initial stages that was manifested as LA enlargement and reduction of fractional shortening and ejection fraction compared to non-diabetic control ZL rats. These effects were accompanied by hypertrophy, elevated myocardial content of TGF*β*1, which promotes the expressions of several fibrotic markers, and interstitial and perivascular collagen deposition, all indicative of cardiac fibrotic remodelling [35]. However, supplementation with the CCB rich-diet alleviated the cardiac structural damages and mitigated the functional abnormalities in the LV of diabetic animals. Similar results have been found in previous reports showing the ability of cocoa flavanols to attenuate cardiac fibrosis during myocardial ischemia [36] and in pressure overload-induced heart failure [37] model mice. In addition, the supplementation with carob extracts decreased the expression of the pro-fibrotic marker TGF*β*1 and arterial vascular fibrosis in mice fed with a high fat/high sucrose diet [24] and in rabbits with dyslipidaemia [38]. Altogether, these results strongly support the protective effect of the CCB rich-diet against myocardial fibrosis, which could have significant therapeutic benefits in the treatment of DCM. More importantly, the combination of CCB with metformin had an additional beneficial effect by completely limiting the fibrotic and the cardiac remodelling in ZDF rats, making the CCB a promising candidate for cardiac co-adjuvant therapy.

At the molecular level, oxidative stress induced by hyperglycaemia is the major pathophysiological mechanism underlying cardiac fibrosis and hypertrophy during the development of DCM [39]. For that reason, both the neutralization of ROS production and the improvement of antioxidant defences may represent an extra protective approach against diabetes-induced oxidative stress in the myocardium. In this sense, the CCB diet proved to be effective in attenuating cardiac oxidative stress by reducing the expression of NOX2 and NOX4 and preventing the generation of ROS and oxidative damage in the LV of diabetic animals. Additionally, levels of the redox-sensitive transcription factor Nrf2 and its downstream antioxidants increased with CCB supplementation, further supporting the antioxidant efficacy of this potentially bioactive food. These results are consistent with several current studies evidencing the ability of certain flavonoids to prevent cardiac oxidative stress through the activation of Nrf2 and the consequent induction of antioxidant enzymes [40,41,42,43]. In particular, we recently found that supplementation with a cocoa rich diet prevented arterial oxidative damage by increasing the levels of Nrf2 and its antioxidant enzymes in the arteries of ZDF diabetic animals [19]. Collectively, these data indicate that CCB may alleviate myocardial fibrosis and the development of DCM in part by increasing Nrf2 levels and reducing oxidative stress in the myocardium. Interestingly, up-regulation of Nrf2 and its downstream antioxidant defences are considered one of the best targets for adjuvant cardiac therapy, especially to prevent DCM [44].

SIRT1 is a histone deacetylase involved in multiple biological processes, that displays a regulatory role in the control of oxidative stress via Nrf2 signalling and, consequently, in the endogenous antioxidant defences status [45]. In the present study, CCB diet increased the levels of SIRT1 that were significantly depleted in the LV of ZDF control rats and reached ZL values in combination with metformin. Enhanced SIRT1 levels due to the flavonoid effect have been observed in other studies using pure flavonoids, such as daidzein [46], curcumin [47], or naringenin [48]. Therefore, the reduced oxidative stress and increased antioxidant activity induced by the mix of flavonoids presented in CCB could be mediated by the sirtuin pathway, as proven in in vitro and in vivo studies [38,46,47,49]. Interestingly, several studies have identified other sirtuins (mainly SIRT3 and SIRT6) to provide beneficial effects in cardiovascular diseases and energy metabolism [50]. Accordingly, a very recent report described the protective effect of the flavone acacetin against cardiac senescence via SIRT1-mediated activation of SIRT6/AMPK signalling pathway [51]. Likewise, the protection of acacetin against vascular hyperglycemic injury was related to SIRT1-mediated activation of SIRT3/AMPK [52]. Moreover, SIRT6 and SIRT3 mutually regulate each other’s activity to protect the heart from developing diabetic cardiomyopathy [53]. Therefore, we cannot rule out the participation of SIRT3 and SIRT6 in the protective effects found in our experimental model.

Persistent oxidative stress can also stimulate the activation of the nuclear transcriptional factor NFκB in diabetic hearts, leading to several inflammatory responses [54]. Above all, NFκB induces the expression of a number of cytokines (TNFα, IL1, IL6), which are involved in the cardiomyocyte hypertrophy [55]. Likewise, NF-κB can activate the TGFβ/Smad2/3 pathway, promoting the expressions of fibrotic markers and thus facilitating the progression of fibrosis in the diabetic heart [9]. In the present study, ZDF diabetic animals showed myocardial inflammation as indicated by leucocyte infiltration (CD45 positive staining), increased NF-κB values and augmented pro-inflammatory cytokines TNFα, IL6, and MCP1. However, CCB supplementation significantly reduced the expression of all these pro-inflammatory mediators, the combination with metformin displaying superior protective effects on myocardial inflammation. These results were consistent with the significant reduction of TGFβ1 and collagen deposition found in the hearts of ZDF diabetic rats treated with CCB, metformin or both. The anti-inflammatory effect of flavonoids in DCM has been previously shown [40,41,43,56]. Especially, reduced levels of cardiac IL-6, MCP-1, TNFα and TGFβ1 in obese mice supplemented with a carob fruit extract have also reported [22]. Moreover, the daily administration of a cocoa extract significantly reduced the levels of inflammatory markers (IL-6 and NF-kB) in the heart of rats exposed to a myocardial ischemia-reperfusion injury [57]. Largely, these findings support the potent anti-inflammatory activity of the cocoa–carob blend, providing an additional cardioprotective effect against DCM. Additionally, it should be considered that NF-κB is a target of SIRT1 [8,58] and may also contribute to the regulation of inflammation.

## 5. Conclusions

The present work illustrates that chronic supplementation of ZDF diabetic rats with a cocoa–carob blend diet rich in flavonoids significantly prevents the development of cardiac hypertrophy and fibrosis, which ultimately alleviates cardiac dysfunction. This protective effect seems to be a consequence of its anti-hyperglycaemic, antioxidant and anti-inflammatory properties. In this sense, the association between all these effects and SIRT1/Nrf2/NFκB pathways is suggested. Notably, the CCB and metformin tandem had a greater response, suggesting additive effects and representing a new strategy to handle DCM.

Overall, our findings highlight the potential of CCB, alone or in adjuvant therapy, for treating DCM. Further clinical studies to confirm these effects in humans would offer a more effective and safe alternative to reduce cardiovascular mortality in T2D subjects.

## Figures and Tables

**Table 1:** Composition of the experimental control and cocoa–carob diets.

Component (g/Kg Dry Weight)	Control	Cocoa–Carob Blend
Casein	140	140
Dextrose	155	155
Sucrose	100	92
Fat	40	40
*t-BHQ* (*tert*-butylhydroquinone)	0.008	0.008
Mineral mix.	35	35
Vitamin mix.	10	10
L-Cys	1.8	1.8
Cholin bitartrate	2.5	2.5
Cellulose	100	44.5
Starch	415.7	379
Cocoa–carob powder	-	100
Energy (KJ/Kg diet)	15048	15048

**Table 2:** Initial and final body weight, food intake and food efficiency of Zucker Lean rats (ZL); Zucker Diabetic rats (ZDF); Zucker Diabetic rats treated with metformin (ZDF (M)); Zucker Diabetic rats fed with a CCB rich-diet (ZDF (CCB)), and Zucker Diabetic rats treated with metformin and fed with a CCB rich-diet (ZDF (CCB + M)). Data represent the means ± SD of 6–8 animals. Means in a row without a common letter differ, *p* < 0.05.

	ZL	ZDF	ZDF (M)	ZDF (CCB)	ZDF (CCB + M)
Initial Body weight (g)	278 ± 9 ^a^	340 ± 11 ^b^	341 ± 20 ^b^	337 ± 26 ^b^	338 ± 23 ^b^
Final Body weight (g)	377 ± 17 ^a^	393 ± 13 ^ab^	423 ± 38 ^b^	384 ± 26 ^ab^	411 ± 30 ^ab^
Body weight gain (g)	98 ± 10 ^a^	55 ± 8 ^b^	93 ± 17 ^a^	63 ± 5 ^b^	74 ± 14 ^c^
Total food intake (g in 12 weeks)	1547 ± 61 ^a^	2491 ± 38 ^b^	2390 ± 44 ^b^	2466 ± 39 ^b^	2372 ± 48 ^b^
Food Efficiency (body weight gain/total food intake) (%)	6.4 ± 0.6 ^a^	2.2 ± 0.3 ^b^	3.9 ± 0.7 ^c^	2.5 ± 0.2 ^bd^	3.1 ± 0.6 ^d^

**Table 3:** Heart weight-to-tibia length ratio (HW/TL), left ventricular end-diastolic dimensions (LVDD), left ventricular end-systolic dimensions (LVSD), septum diastolic diameter (IVSD), LV posterior wall diastolic diameter (LVPWD), left atrium diameter (LA) and aorta diameter (Ao) of Zucker Lean rats (ZL); Zucker Diabetic rats (ZDF); Zucker Diabetic rats treated with metformin (ZDF (M)); Zucker Diabetic rats fed with a CCB rich-diet (ZDF (CCB)), and Zucker Diabetic rats treated with metformin and fed with a CCB rich-diet (ZDF (CCB + M)). Data represent the means ± SD of 6–8 animals. Means in a row without a common letter differ, *p* < 0.05.

	ZL	ZDF	ZDF (M)	ZDF (CCB)	ZDF (CCB + M)
HW/TL (g/cm)	0.32 ± 0.02 ^a^	0.32 ± 0.01 ^a^	0.35 ± 0.03 ^a^	0.26 ± 0.01 ^b^	0.31 ± 0.01 ^a^
LVDD (cm)	0.73 ± 0.05 ^a^	0.76 ± 0.05 ^a^	0.75 ± 0.08 ^a^	0.65 ± 0.05 ^a^	0.68 ± 0.13 ^a^
LVPWD (cm)	0.22 ± 0.08 ^a^	0.20 ±0.05 ^a^	0.20 ± 0.05 ^a^	0.19 ± 0.04 ^a^	0.21 ± 0.04 ^a^
LVSD (cm)	0.40 ± 0.06 ^a^	0.46 ± 0.05 ^a^	0.38 ± 0.07 ^a^	0.39 ± 0.06 ^a^	0.39 ± 0.10 ^a^
IVSD (cm)	0.13 ± 0.05 ^a^	0.14 ± 0.05 ^a^	0.15 ± 0.05 ^a^	0.14 ± 0.05 ^a^	0.15 ± 0.05 ^a^
Ao diameter (cm)	0.33 ± 0.05 ^a^	0.23 ± 0.05 ^b^	0.32 ± 0.04 ^ab^	0.28 ± 0.08 ^ab^	0.28 ± 0.04 ^ab^
LA diameter (cm)	0.37 ± 0.08 ^a^	0.57 ± 0.08 ^b^	0.50 ± 0.05 ^b^	0.43 ± 0.07 ^ab^	0.49 ± 0.04 ^ab^

## Data Availability

The data presented in this study are available in the article.
